# Health literacy in adolescents and young adults in Benin: French translation and validation of the health literacy measure for adolescents (HELMA)

**DOI:** 10.3389/fpsyg.2024.1428434

**Published:** 2024-09-18

**Authors:** Bonaventure G. Ikediashi, Cristina Ehrmann, Gisela Michel

**Affiliations:** ^1^Faculty of Health Sciences and Medicine, University of Lucerne, Lucerne, Switzerland; ^2^Swiss School of Public Health, Zürich, Switzerland; ^3^Swiss Paraplegic Research, Nottwil, Switzerland

**Keywords:** health literacy, adolescents and young adult (AYA), translation and validation, psychometric properties, Benin

## Abstract

**Background:**

The Health Literacy Measure for Adolescents (HELMA) is a self-assessment validated tool used to measure health literacy in adolescents. This study aims to evaluate the psychometric properties of the French translation of the Health Literacy Measure for Adolescents (F-HELMA).

**Methods:**

The HELMA questionnaire was translated according to the World Health Organization's (WHO) recommendation for translation and adaptation of instruments. It was pre-tested with 30 students. Subsequently, 495 adolescents and young adults from five senior secondary high schools in Benin completed the questionnaire. A sample of 44 participants completed the questionnaire twice over a 2-week period to determine the test-retest reliability. Construct validity was evaluated using confirmatory factor analysis (CFA) and convergent validity was analyzed the Health Literacy Assessment Tool.

**Results and discussion:**

The F-HELMA—French translation of the Health Literacy Measure for Adolescents, showed moderate to good psychometric properties. CFA showed good fit indices for a seven-factor model. Reliability figures fell within an acceptable range; Cronbach's alpha ranged from 0.64 (moderate) to 0.89 (good) across the different subscales, and the intraclass coefficient (ICC) ranged from 0.82 to 0.96, indicating good test-retest reliability. Pearson correlation with HLAT-8 showed good convergent validity (*r* = 0.54, *p* < 0.001). This study provides support for the use of the F-HELMA, as a valid and reliable instrument to measure health literacy in adolescents and young adults in West African French speaking countries.

## Introduction

Health literacy is an important determinant of health. It influences both individual and public health outcomes (Sørensen et al., 2012; Nutbeam and Lloyd, 2021; Manganello and Hadley, 2022; Flores et al., 2023). Defined as “the cognitive and social skills that enable individuals to access, understand, and use information in ways that promote and maintain good health” (Nutbeam, 2000), health literacy is crucial for informed health decision-making (Peerson and Saunders, 2009; Woudstra et al., 2018; Harzheim et al., 2023). Particularly for adolescents and young adults at critical developmental stages, enhancing health literacy is crucial as they form habits impacting long-term health outcomes and navigate the complex health information landscape (Fleary et al., 2018). Research shows that health literacy can directly improve health outcomes across various demographics (Berkman et al., 2011), and indirectly mitigates the impact of socioeconomic disparities by enhancing health behaviors and improving access to healthcare services (Sørensen et al., 2012).

Several health literacy instruments have been developed over the last decade to assess different components of health literacy in an adolescent population (Guo et al., 2018). The Rapid Estimate of Adolescent Literacy in Medicine (REALM-Teen) and the Short Test of Functional Health Literacy in Adults (S-TOFHLA) are some of the commonly used health literacy instruments for this population (Ylitalo et al., 2018). The REALM-Teen assesses health literacy based on participants' ability to read and pronounce a set of words (Manganello et al., 2017). While this instrument is recognized for its strong reliability and short duration of administration, it uses a limited approach measuring only one dimension of health literacy (Guo et al., 2018) and neglects other important aspects of the definition of health literacy, such as the ability to use health information (Dumenci et al., 2014). The S-TOFHLA has a numeracy component in addition to two prose passages used to determine functional health literacy. While this instrument equally has good reliability and validity (Baker et al., 1999), it has been reported that it underestimates inadequate health literacy levels (Housten et al., 2018).

The Health Literacy Measure for Adolescents (HELMA) is a comprehensive instrument designed around Nutbeam's multifaceted health literacy model (Ghanbari et al., 2016), which distinguishes between functional, interactive, and critical dimensions of health literacy (Nutbeam, 2000). The HELMA operationalizes these dimensions through a 44-item questionnaire that spans eight domains: access, reading, understanding, appraisal, use, communication, self-efficacy, and numeracy. Functional health literacy describes the basic skills of reading and comprehension of health information. This dimension assessed by the HELMA through the domains of reading, understanding, and numeracy provides the foundation for making informed decisions. Interactive health literacy goes a step further by evaluating the adolescent's capacity to apply health information in everyday life, fostering effective communication and social interaction. It is captured in the HELMA through the domains of use and communication, which assess the ability to discuss health concerns and interpret messages in dynamic social settings. Critical health literacy, the most advanced dimension, is explored through the domains of appraisal and use. It reflects the capacity to “critically analyze information and use it to exert control over life events and situations.” The domain of self–efficacy measures an individual's belief and confidence to carry out certain health behaviors (Lawrance and McLeroy, 1986), and has been noted as an important element of health literacy, particularly relevant to an adolescent population (Massey et al., 2012).

In the study of health literacy, it is essential to consider the cultural context in which individuals develop and interact. Cultural norms, values, and practices significantly influence how young people perceive and use health information (Leijen and van Herk, 2021; Efthymiou et al., 2023), affecting the effectiveness of health literacy tools tailored to their needs. It is, therefore, necessary to have tools that are culturally sensitive and adaptable to different settings. The HELMA was originally developed and adapted for use in an Iranian adolescent population and adjusted for a comprehensive approach to measuring health literacy and good psychometric properties. To date, there is no published research evaluating health literacy using a standardized instrument or assessing health literacy instruments in the context of Benin. This study aims to: (1) evaluate the psychometric properties of a translated French version of the HELMA (F-HELMA) and (2) examine associations between self-reported health status, socio-demographic characteristics, and the F-HELMA in Benin, a low-resource country in Western Africa.

## Materials and methods

### Translation

Translation of the HELMA questionnaire was done in accordance with the World Health Organization's recommendations for translation and adaptation of instruments (World Health Organization, 2021). The HELMA questionnaire was translated from English to French by two independent native French speakers (one, a registered nurse in Switzerland and the other, an MSc in Communication Sciences). They were instructed to translate the HELMA, taking the meaning of questions into consideration. Afterwards, both translated versions were compared and reviewed with input from another French native speaker (with a BSc in Psychology and an MSc in Health Sciences) to reach a draft of the French version. This draft version was then back-translated from French to English by a fourth independent translator (with a BA in Linguistics) and compared with the original English version to check that there was semantic value and to establish the final version of the questionnaire. The comparisons established that the final French version of the questionnaire provided the same semantic value as the original English questionnaire. There were only few minor differences between the original HELMA questionnaire and the back-translation document. The French version of the HELMA (F-HELMA), was subsequently administered to 30 students for a pilot pre-test. This was done to check for any difficulties that could be encountered, the clarity of the instructions provided, and any understanding or misunderstandings of the questions. This was accompanied by verbal feedback obtained from the students to get their impressions such as any difficulty, length of time needed to complete the questionnaire, and the layout of the questionnaire. There were only few minor alterations in wording based on the feedback provided.

### Sample and procedure

We used a convenience sample of five schools in the Atlantic Littoral Department in Benin. Participants were recruited from the senior classes (i.e., first, second and final years of senior secondary school). The questionnaires were administered to 585 students, of whom 533 students returned the questionnaires. After excluding 38 questionnaires because they were either not completed at all or largely incomplete, we finally included 495 valid questionnaires in the analysis. This number is in line with the rule of thumb recommendation used by the authors of the HELMA questionnaire, which stated that 470 respondents are sufficient a sample size (Nunnally, 1978).

### Questionnaires

#### HELMA

The HELMA consists of eight domains with a varying number of items per domain as follows: (I) Access: five items; (II) Reading: five items; (III) Understanding: 10 items; (IV) Appraisal: five items; (V) Use: four items; (VI) Communication: eight items; (VII) Self-efficacy: four items; and (VIII) Numeracy: three items. Each item is scored on a scale of 1–5, with 1 indicating “never” (a low score), 2 “rarely,” 3 “sometimes,” 4 “usually,” and 5 indicating “always” (a high score). However, the scoring for the numeracy scale differs; it is determined by a mathematical calculation with a score of 1 assigned for incorrect answers and 5 for correct answers (see Supplementary Appendix 2). The raw scores were summed up and linearly transferred to a score from 0 to 100 to determine the total health literacy score. HELMA scores are classified into four categories: inadequate (0–50.0), problematic (50.1–66.0), sufficient (66.1–84.0) and excellent (84.1–100). The eight domains of the HELMA constitute an eight factor model proposed by the authors of the HELMA (Ghanbari et al., 2016).

#### Health literacy assessment tool

In addition to the HELMA questionnaire, all the participants completed a validated measure of health literacy in an adolescent population—the Health Literacy Assessment Tool (HLAT-8). The HLAT-8 was first developed and used to measure health literacy in a young adult population in Switzerland in 2018 (Abel et al., 2015). It consists of eight questions and assesses functional, interactive, and critical domains of health literacy. This questionnaire was also administered to the participants to assess the convergent validity properties of the HELMA instrument.

#### Health status and socio-demographic characteristics

Finally, participants provided information on their self-reported health status by responding to a single-item indicator (“How would you rate your health?”: Very good, Good, Moderate, Bad or Very bad). The responses were later recoded, collapsing “Moderate,” “Bad,” and “Very bad” into a new category termed “Somewhat,” because there were very few recorded responses for “Bad” and “Very Bad” while “Very good” and “Good” were retained as separate categories. In addition, socio-demographic characteristics namely gender, year of study, and study track (Art, Sciences or Technical) were collected. We used parental education levels (primary, secondary, and tertiary) and parental employment status (employed, unemployed self-employed or retired) as proxy for socio-economic status.

### Analyses

All statistical analyses were carried out using the R Statistical Software (version 4.0.2, R Core Team, 2020) and Stata Statistical Software (Release 18. College Station, TX). Descriptive statistics were carried out to give an overview of the responses. For items with missing data, the least value of 1 for the HELMA questionnaire was inputted and 0 for the HLAT-8 questionnaire. To assess the psychometric properties of the F-HELMA questionnaire, we employed a series of analyses. We evaluated internal consistency by computing Cronbach's alpha. In addition, we computed the Intraclass Correlation Coefficient (ICC) on a sample of 44 students who completed the F-HELMA again within a 2-week period to assess test-retest reliability. In addition, we computed the “alpha if item deleted statistic,” to assess whether deleting any item would improve the Cronbach's alpha. Considering the structure of the HELMA questionnaire was known, and the research question sought to test a predetermined model which best fit the F-HELMA data, an exploratory factor analyses was not conducted (Suhr, 2006; Brown, 2015). We conducted Confirmatory Factor Analysis (CFA) using the Lavaan R package to assess the model for the eight scales of the HELMA. For estimation, we employed the Weighted Least Squares Mean and Variance Adjusted (WLSMV) method, suitable for the ordinal nature of our data and when multivariate normality is not assumed (Han, 2022). To assess the model fit in our study, we employed various fit indices, including the Comparative Fit Index (CFI), Tucker Lewis Index (TLI), Root Mean Square Error of Approximation (RMSEA), and the Standardized Root Mean Square Residual (SRMR). Consistent with established guidelines (Hu and Bentler, 1999), values exceeding 0.90 for both CFI and TLI and values <0.08 for both RMSEA and SRMR were considered indicative of an acceptable fit. The RMSEA and SRMR function as absolute fit indices, quantifying the disparity between a hypothesized model and an ideal, perfect model. In contrast, CFI and TLI serve as incremental fit indices, evaluating the improvement in fit by comparing the hypothesized model against a baseline model (Xia and Yang, 2019). To assess the relationships between the latent constructs of the F-HELMA, we extracted the latent variable correlation matrix from the fitted CFA model using the lavInspect function in the lavaan package within the R software. Finally, we examined convergent validity by assessing correlations between the F-HELMA questionnaire (excluding the numeracy subscale) and the HLAT-8 questionnaire.

## Results

### Participants characteristics

The sample consisted of 495 adolescents and young adults (49.3% females) in the final three years of senior secondary schooling who completed the F-HELMA, HLAT-8, and socio-demographic questionnaires. The average age was 16.5 years (range 14–25 years). Most participants were in the Sciences study track (67.9%), while 24.7% were in the Arts study track and 5.4% the Technical study track. Regarding parental educational level, most of the fathers (40.6% secondary, 30.5% tertiary level) and around half of the mothers (36.4.% secondary, 14.1% tertiary level) had higher education. In terms of employment status, most parents were either employed (fathers: 39.8%, mothers: 20.0%) or self-employed (fathers: 43.6 %, mothers: 66.6 %). The health status reported by most participants was either “Very good” (26.2%) or “Good” (38.0%). Participants' characteristics are shown in Table 1.

**Table 1:** Demographic characteristics of participants.

	**Mean**	**SD**
	***N***	**%**
Age (years)	16.5	3.2
**Gender**		
Male	240	48.5
Female	244	49.3
Missing	11	2.2
**Year of study**		
First year	146	29.5
Second year	189	38.2
Final year	146	29.5
Missing	14	2.8
**Study track**		
Arts	122	24.7
Sciences	336	67.9
Technical	26	5.3
Missing	11	2.2
**Father education**		
University	151	30.5
Secondary	201	40.6
Primary	79	16
Missing	64	12.9
**Mother education**		
University	70	14.1
Secondary	180	36.4
Primary	173	35
Missing	72	14.6
**Father employment**		
Employee	198	40
Self-employed	214	43.2
Unemployed	8	1.6
Retired	37	7.5
Missing	38	7.7
**Mother employment**		
Employed	99	20
Self-employed	329	66.5
Unemployed	26	5.3
Retired	5	1
Missing	36	7.3
**Health status**		
Very good	128	26
Good	189	38.2
	***N***	**%**
Somewhat	120	24.2
Poor	13	2.6
Very poor	1	0.2
Missing	44	8.9

SD, standard deviation.

Missing, the answer was not completed.

### Psychometric characteristics

#### Reliability

Cronbach's alpha for the seven subscales used in the final model ranged from moderate (numeracy: 0.64; reading: 0.67; appraisal: 0.68; access: 0.69; use: 0.68) to good (self-efficacy: 0.72; communication: 0.77; total F-HELMA scale: 0.89; Table 2). The deletion of any item did not result in any further improvements to internal consistency (Supplementary Appendix 1). The F-HELMA subscales (seven factor model) showed a good to excellent intra-class coefficient (ICC) ranging from 0.82 (reading) to 0.90 (understanding) and 0.96 for the total F-HELMA scale (Table 2).

**Table 2:** Internal consistency and intraclass coefficient of the F-HELMA.

**Scale**	**Number of items**	**Cronbach's alpha (*n* = 495)**	**ICC (*n* = 44)**
Self-efficacy	4	0.721	0.85
Access	5	0.685	0.83
Read	5	0.671	0.82
Understanding	10	0.836	0.90
Appraisal	5	0.681	0.86
Use	4	0.684	0.84
Communication	8	0.774	0.89
Numeracy	3	0.638	0.78
Approach	14	0.710	0.91
Total F-Helma (eight-factor model)	44	0.891	0.96
Total F-Helma (seven-factor model)	41	0.905	

ICC, intraclass coefficient.

Eight-factor model includes self-efficacy, access, read, understand, appraisal, use, communication, and numeracy; Numeracy is dropped in the seven factor model.

#### Confirmatory factor analysis

The data in our study did not conform to the assumptions of a normal distribution. Both the Henze–Zirkler test (*p* = 0), used to assess multivariate normality, and the Shapiro–Wilk test, used to evaluate univariate normality (*p* < 0.001), indicated a non-normal distribution. Consequently, we adjusted our analysis using the WLSMV to account for these distributional characteristics. To investigate whether the previously established eight-factor structure of the HELMA was applicable to the French-translated version, we initially fitted an eight-factor model (i.e., consisting of the eight domains self-efficacy, access, reading, understanding, appraisal, use, communication, and numeracy). Subsequently, we did not include the numeracy scale because it has a different scoring system (1, for wrong response and 5, for correct response). The results of the confirmatory factor analysis indicated a good fit for the seven-factor model of the F-HELMA scales, supported by the fit indices: chi-square χ^2^ = 6499.418, df = 820, *p* < 0.000; CFI (0.958), TLI (0.954), RMSEA = 0.044 (90% CI, 0.040; 0.048), and SMMR (0.054). Standardized factor loadings ranged from 0.412 to 0.913, as presented in Table 3. There were only five items that had factor loadings lower than 0.600. Among the items, the one with the lowest factor loading was, “When shopping, I choose food based on its nutrition facts (e.g., amount of energy, sugar, protein, etc.) written on the packaging” (0.412) on the use scale (see Supplementary Appendix 1).

**Table 3:** Results of confirmatory factor analysis of the F-HELMA.

**Model**	**χ^2^ value**	**df**	**CFI^a^**	**TFI^b^**	**SRMR**	**RMSEA (90% CI)**	***p*-Value**
Eight factor model^c^	6,778.500	946	0.957	0.954	0.053	0.041 (0.037, 0.045)	<0.001
Seven factor model^d^	6,499.418	820	0.958	0.954	0.054	0.039 (0.040, 0.048)	<0.001

^a, b^Robust measures reported due to non-normally distributed data.

χ^2^, chi-square; df, degrees of freedom; CFI, comparative fit index; TFI tucker-lewis index; SRMR, standardized root mean square RMSEA root mean square error of approximation; CI, confidence interval.

^c^Eight-factor model includes self-efficacy, access, read, understand, appraisal, use, communication, and numeracy; ^d^numeracy is dropped in the seven factor model.

#### Convergent validity

The correlation between the HLAT-8 score (Spearman's correlation) and the total F-HELMA score (excluding the numeracy scale) was moderate (*r* = 0.54, *p* < 0.001), indicating convergent validity. The correlation with the 8-Factor F-HELMA score (including the numeracy) was slightly lower (*r* = 0.44, *p* < 0.001).

#### Factor correlations

The inter-factor correlations between the seven F-HELMA scales ranged between 0.37 and 0.73 (Table 4). The highest correlation was found between Self-efficacy and understanding (*r* = 0.73). The lowest correlations were found between self-efficacy and reading (*r* = 0.34), and self-efficacy and appraisal (*r* = 0.37).

**Table 4:** Correlation between latent variables of the seven factor model of the F-HELMA.

	**1**	**2**	**3**	**4**	**5**	**6**	**7**
1 Self-efficacy	1.00						
2 Access	0.46	1.00					
3 Reading	0.34	0.52	1.00				
4 Understanding	0.73	0.47	0.48	1.00			
5 Appraisal	0.37	0.53	0.49	0.57	1.00		
6 Use	0.56	0.49	0.45	0.60	0.57	1.00	
7 Communication	0.56	0.48	0.46	0.62	0.49	0.66	1.00

Correlations between latent variables are based on estimates from the confirmatory factor analysis using the DWLS estimator. These are polychoric correlations computed as part of the CFA model estimation; p-value for all correlation coefficients p <0.001.

#### Distribution of health literacy scores by socio-demographic factors and health status

Total F-HELMA score was associated with educational level of mothers, the academic majors pursued, and the self-reported health status. However, there were no significant differences in total F-HELMA scores concerning age, gender, fathers' education, and fathers' occupation (Table 5).

**Table 5:** Scores of the total F-HELMA scale by demographic characteristics.

	***N***	**Mean**	**SD**	**Test statistic**	***p*-Value**
Age^a^	492	54.82	3.27	0.038	0.492
**Gender** ^b^					
Male	240	54.08	14.15	0.461	0.139
Female	244	56.00	14.36		
**Majors** ^c^					
Art (ref)	122	51.68	12.70	**5.771**	**0.004** ^*^
Sciences	336	**56.03**	**14.51** ^*^		
Technical	26	**59.25**	**14.17** ^*^		
**Year of study** ^c^					
First year (ref)	146	59.21	12.87	**9.068**	**<0.001** ^**^
Second year	189	**53.04**	**14.35** ^**^		
Final year	146	**53.74**	**14.67** ^*^		
**Father education** ^c^					
Primary (ref)	151	54.52	15.27	0.239	0.788
Secondary	201	55.48	13.20		
Tertiary	79	54.41	15.74		
**Mother education** ^c^					
Primary (ref)	70	50.67	14.44	**7.105**	**0.01**
Secondary	180	**57.67**	**13.85** ^*^		
Tertiary	173	53.67	14.48		
**Father employment** ^c^					
Employee (ref)	198	54.64	14.70	2.459	0.069
Self-employed	214	56.82	13.71		
Unemployed	8	44.85	14.45		
Retired	37	53.01	15.94		
**Mother employment** ^c^					
Employee (ref)	99	51.28	14.97	**4.956**	**0.005** ^*^
Self-employed	329	**56.92**	**13.96** ^*^		
Unemployed	26	50.87	14.66		
Retired	5	56.39	13.05		
**Health status** ^c^					
Somewhat (ref)	134	52.53	13.48	**4.347**	**0.014** ^*^
Good	189	**57.28**	**14.66** ^*^		
Very good	128	55.40	14.70		

^a^Bivariate analysis applied.

^b^Independent t-test.

^c^One way ANOVA.

^*^p <0.05.

^**^p <0.001.

Significant values are in bold.

SD, standard deviation.

## Discussion

In this study, we report the findings from our validation of the French translation of the HELMA (F-HELMA) questionnaire in Benin. Using data collected from a sample of young students aged 14–25 years. Our results suggest a moderate replication of the original HELMA. This is evidenced by good CFA fit indices, moderate to good Cronbach's alpha, and good to excellent intraclass correlation coefficients. The F-HELMA is thus suitable for use in assessing health literacy among adolescents and young adult in the context of Benin.

The reliability of an instrument can be measured by its internal consistency which indicates the extent to which the different dimensions of the instrument measure the same construct (Revicki, 2014). In the case of the F-HELMA, reliability analyses were conducted to determine if the eight different domains all measure the same construct of health literacy. The total score of F-HELMA displayed good internal consistency, with a Cronbach's alpha of 0.89, while the subscales also had values that can be considered satisfactory (access, reading, appraisal, and use) or good (communication, self-efficacy, and understanding). Overall, these values were comparable to what was obtained by the authors of the original HELMA questionnaire (Housten et al., 2018). However, they had obtained an excellent Cronbach alpha of 0.93 for the total HELMA scale, whereas our data resulted in a Cronbach alpha of 0.89 which is considered good. Another study which evaluated the psychometric properties of the HELMA instrument found a slightly lower Cronbach alpha of 0.74 for the overall HELMA scale among youths from Malaysia and Sri Lanka (Vashe et al., 2022). The stability test, as measured by the ICC is another measure of the reliability properties of the instrument, demonstrating how an instrument behaves with repeated administrations at different times (Terwee et al., 2007). The ICC values indicated high test-retest reliability for all subscales and the total scale. These results suggest strong agreement between measurements taken on two separate occasions, supporting the reliability of the F-HELMA.

The best fitting model for our data according to the CFA was the seven-factor model, which excluded the numeracy scale, although the fit statistics for both models tested were good. A previous study conducted in Malaysia suggested excluding item 41 “I talk to friends about avoiding risky behavior (e.g., smoking, hookah, drugs),” from the scale (Vashe et al., 2022). The authors concluded that this question might have been avoided in the South Asian context. Based on the internal consistency results, deleting this item or any other item did not result to an improved Cronbach alpha. When the CFA was conducted with the eight-factor model, it resulted in factor loadings for the numeracy scale >1. Furthermore, the results of the factor correlations differed significantly from the other factors. This unusual finding can be attributed to the unique scoring system of the numeracy sub-scale which uses a binary scoring system (1 for wrong and 5 for correct) which is different from the ordinal Likert scales used for the other subscales. Binary data, with its limited variability and lack of nuance compared to ordinal data, can result in a loss of information. Furthermore, the assumption of equidistant intervals inherent in ordinal data does not apply to binary data (Suárez-García et al., 2024). Unusual factor loadings suggest that the scale may not be measuring the same underlying construct as other subscales. However, numeracy is one important aspect of health literacy (Weiss et al., 2005) and thus complements the other dimensions. We also assume that the students did not respond to the numeracy items in the same manner as they did with the other items. This could be due to lack of familiarity or comfort with mathematical tasks. The varied response might explain the unusual pattern in factor loadings and correlations observed. To make sure the numeracy items better match the overall construct of health literacy as measured in this context, further evaluation or adjustment of the items may be necessary. In evaluating the convergent validity of the F-HELMA, we anticipated a correlation with the HLAT-8, given that both instruments measure the construct of health literacy. We expected that the scores from these two tools would be related, considering they both measure similar constructs of health literacy. Our findings support this expectation, as evidenced by a significant, moderate positive correlation between the total F-HELMA score—excluding the numeracy subscale and the HLAT-8 score.

One of the key strengths of the HELMA instrument is that it takes a comprehensive approach to health literacy. Except for self-efficacy and understanding, the small to moderate correlations observed between the other subscales of the F-HELMA suggest that these subscales are related but not identical, and therefore measure different aspects of health literacy. This further supports the multidimensionality of the F-HELMA. Health literacy is not a single, homogeneous construct but comprises several related dimensions (Nutbeam, 2000). The high correlations found between self-efficacy and understanding suggest that belief in one's ability to carry out certain health behaviors may be linked to their understanding of health information.

Overall, higher health literacy scores were associated with a better health status, which could be explained by positive information-seeking behaviors and taking actions that improve health (Baker et al., 1999; Chang, 2011). As expected, we found associations between the study track, and the F-HELMA scores. We believe the reason can be linked to an interest in the sciences. Senior secondary school students in Benin are assigned to different study tracks based on interest in the study track as well as performance in the qualifying examinations. Thus, those who end up in the sciences would have performed above average and would be more likely to have more interest in the sciences. Students in the sciences and technical study track scored higher than their counterparts who studied the arts. Compared to students in the first year, those in the second and third year recorded a lower mean health literacy score. The reasons for this, remains unclear, as one would expect that those in the higher years would have more health knowledge and abilities in general. We can hypothesize that this may be that the first-year students were overestimating their abilities. In this study, we did not find any associations between gender or age and the F-HELMA score. Other studies in the literature have shown varying results. For example, while one study conducted with college students reported no significant associations between gender and health literacy levels (Ickes and Cottrell, 2010), another study reported contrasting findings (Uysal et al., 2020; Chu-Ko et al., 2021). One possible explanation for the lack of association between gender and F-HELMA scores could be that the instrument is not sensitive to detect gender differences.

In addition, age did not appear to be a significant factor in our study, however, another study conducted, identified an association between health literacy and age (Vashe et al., 2022). We believe age was not so important compared to the study track and year of study. These aspects better reflect participants' level of education and may influence their health literacy. Our findings revealed that health literacy scores did not differ according to the father's employment status. Similarly, no differences were observed in health literacy scores by fathers' education levels. However, we observed a statistically significant association with mothers' education and employment status. Participants with mothers who had a higher level of education and were self-employed achieved higher scores. These findings are consistent with prior research showing the importance of parental education for children's health literacy (Davis-Kean, 2005). In the context of Benin, this trend may be attributed to the time self-employed mothers spend with their children, potentially influencing their health literacy.

Although the validation of the F-HELMA provides new information about health literacy of adolescents and young adults in Benin, we acknowledge that the methodology used in our study has certain limitations. Perception-based instruments like the HELMA, are useful for assessing self-reported health literacy, however they may not fully reflect the respondents' true competencies. This is because individuals might either underestimate or overestimate their health literacy based on personal bias or how they interpret the questions on the questionnaire. Such biases, which have been reported in previous research (Japelj and Horvat, 2023), suggest that the results obtained might reflect ‘perceived' rather than ‘actual' levels of health literacy. Another limitation is that we used the HLAT-8 to measure convergent validity, even though it does not include a numeracy measure. Therefore, the interpretation of convergent validity is limited to the F-HELMA scale, excluding the numeracy subscale. Future validation studies of the HELMA should consider examining convergent validity with a health literacy measure that includes a component of numeracy.

Furthermore, the study was conducted in an urban area and may not be generalized to the entire adolescent and young adult population of Benin. We acknowledge that this setting may not adequately capture the diverse experiences and health literacy levels obtainable in other parts of the country where cultural background, French language proficiency, access to education and healthcare differs. These differences can impact how adolescents and young adults in non-rural areas will respond to some of the questions of the questionnaire. Finally, we used a convenience sample of students who might have refused to participate. It is possible that those who decided not to participate were those with a lower health literacy level. This could lead to an overestimation of the overall health literacy in the sample. We specifically targeted adolescents and young adults aged 14–25 years, which encompasses the age group of the final 3 years of secondary school. Despite these limitations, our study demonstrates that the F-HELMA can be used in a school setting. We were able to include a large sample, offering insights into the health literacy levels in an urban setting in Benin. Our study sample shares a similar demographic profile to the population reported in the study that reported the development of the HELMA. Furthermore, we also evaluated the convergent validity of the instrument which has not previously been evaluated. Finally, we expanded the demographic range from the original 18 years, as reported by the authors, to 25 years. This extension allows for the inclusion of a young adult population, indicating that HELMA is applicable to this age group.

## Conclusion

We show that the F-HELMA is a reliable tool for assessing health literacy among adolescents and young adults in Benin, demonstrating good fit indices from the confirmatory analysis and moderate to good reliability scores from the Cronbach's alpha and intraclass correlation coefficients and immoderate convergent validity with the HLAT-8. This supports its use in educational settings and provides a useful first step for its application in other settings. Developing a shorter version of the F-HELMA could enhance its suitability for settings such as hospitals, where extensive assessments are not practical due to time constraints. In such contexts, a shorter questionnaire would facilitate routine health literacy screenings, which can help identify individuals at risk of adverse health outcomes linked to inadequate health literacy. The CFA of the eight-factor model showed unusual factor loadings >1 for the numeracy subscale and significant differences in correlations between subscales compared to the other factors. Revision and possible modification of items within the numeracy scale may therefore be necessary to better reflect the overall health literacy construct. Further research into the relationship between health literacy and health outcomes can inform programs and policies to improve health literacy.

## Data Availability

The raw data supporting the conclusions of this article will be made available by the authors, without undue reservation.
