# Supplementary material for: Health literacy in adolescents and young adults in Benin: French translation and validation of the health literacy measure for adolescents (HELMA)
Source: Front Psychol. 2024 Sep 18;15:1428434. doi: 10.3389/fpsyg.2024.1428434 (PMC11445019; doi:10.3389/fpsyg.2024.1428434)
Supplement: Supplementary file 1 [file Data_Sheet_1.docx]

**SUPPLEMENTARY MATERIAL**

**APPENDIX 1: Factor loadings of the F-HELMA; Cronbach’s alpha and Intraclass Coefficient Mean F-HELMA, HLAT scores & Cronbach’s alpha.**

| **Items** | **Factor Loading** | **Item-total correlation** | **Item-remainder correlation** | **Alpha if deleted** | **Cronbach’s alpha (n=495)** |
| --- | --- | --- | --- | --- | --- |
| **Self-Efficacy** |  |  |  |  | 0.72 |
| 1. I try to get more information about health as much as possible | 0.862 | 0.74 | 0.61 | 0.66 |  |
| 2. I am able to find health information that I need | 0.841 | 0.79 | 0.71 | 0.61 |  |
| 3. When ill or facing health problems, I can get the necessary information I need | 0.751 | 0.72 | 0.56 | 0.68 |  |
| 4. I am able to ask others about health information that I need | 0.747 | 0.71 | 0.54 | 0.69 |  |
| **Access -** |  |  |  |  | 0.69 |
| 5. I am able to access information about the healthy diet that is appropriate for my age group | 0.800 | 0.72 | 0.62 | 0.51 |  |
| 6. I am able to access information about the physical activity appropriate for my age group | 0.803 | 0.74 | 0.65 | 0.48 |  |
| 7. I am able to access information about the proper care required for my skin and hair that is appropriate for my age group | 0.664 |  |  |  |  |
| 8. I am able to access information about mental health appropriate for my age group | 0.616 | 0.68 | 0.47 | 0.57 |  |
| 9. I am able to find useful resources about health Information on the Internet | 0.700 | 0.60 | 0.35 | 0.64 |  |
| **Reading -** |  |  |  |  | 0.67 |
| 10. I can read brochures on prescribed medicine | 0.743 | 0.72 | 0.65 | 0.58 |  |
| 11. I can easily read educational brochures about nutritional issues | 0.785 | 0.62 | 0.48 | 0.65 |  |
| 12. I can easily read brochures/fact sheets about disease prevention (e.g. anaemia, osteoporosis, respiratory infections, etc.) | 0.773 | 0.72 | 0.63 | 0.57 |  |
| 13. I can easily read health information materials in magazines and newspapers | 0.513 | 0.62 | 0.47 | 0.64 |  |
| 14. I can easily read health information materials on the Internet (e.g. websites) | 0.618 | 0.60 | 0.43 | 0.65 |  |
| **Understanding** |  |  |  |  | 0.84 |
| 15. I can easily understand the meaning of the signs used in hospitals and medical centres | 0.723 | 0.58 | 0.51 | 0.83 |  |
| 16. I can understand most things I hear about health | 0.767 | 0.70 | 0.67 | 0.81 |  |
| 17. I can easily understand the content of health information that I find | 0.701 | 0.63 | 0.59 | 0.82 |  |
| 18. I can easily understand my doctor’s instructions and recommendations (e.g. prescriptions) | 0.897 | 0.72 | 0.69 | 0.81 |  |
| 19. I can easily understand information about medications – usage, side effects and warnings | 0.840 | 0.70 | 0.66 | 0.81 |  |
| 20. I can easily understand the nutrition facts on food packages | 0.630 | 0.60 | 0.54 | 0.82 |  |
| 21. I can understand the information and recommendations about proper nutrition for adolescents in the media (e.g. radio, TV, internet, etc.) | 0.731 | 0.63 | 0.57 | 0.82 |  |
| 22. I can understand the information and warnings provided by the media (e.g. radio, TV, internet, etc.) about tobacco, drug abuse and risky behaviours | 0.678 | 0.66 | 0.61 | 0.82 |  |
| 23. I can understand the information and recommendations about health and illness in the media | 0.541 | 0.58 | 0.53 | 0.83 |  |
| 24. I can understand the recommendations on prevention of accidents and injuries | 0.516 | 0.56 | 0.48 | 0.83 |  |
| **Appraisal** |  |  |  |  | 0.68 |
| 25. When faced with new health information, I can judge its accuracy | 0.546 | 0.63 | 0.46 | 0.65 |  |
| 26. I would compare the data obtained from various sources | 0.679 | 0.67 | 0.52 | 0.64 |  |
| 27. When dealing with conflicting information about health issues, I can recognize the correct information | 0.627 | 0.71 | 0.64 | 0.59 |  |
| 28. I have the ability to judge which resources I can trust | 0.632 | 0.69 | 0.59 | 0.61 |  |
| 29. When dealing with nutritional information I can choose the right information | 0.707 | 0.62 | 0.46 | 0.66 |  |
| **Use - Alpha** |  |  |  |  | 0.68 |
| 30. When shopping, I choose food based on its nutrition facts (e.g. amount of energy, sugar, protein, etc.) written on the packaging | 0.412 | 0.65 | 0.47 | 0.66 |  |
| 31. I try to choose foods without preservatives | 0.792 | 0.75 | 0.58 | 0.61 |  |
| 32. I try to apply what I have learned about health issues in my everyday life | 0.913 | 0.76 | 0.67 | 0.57 |  |
| 33. I try to keep my body weight in balance | 0.827 | 0.71 | 0.57 | 0.63 |  |
| **Communicate** |  |  |  |  | 0.77 |
| 34. I can discuss my concerns relating to health issues with health providers | 0.734 | 0.59 | 0.51 | 0.76 |  |
| 35. When visiting a doctor or health provider I am able to give him/her all of my necessary personal information | 0.829 | 0.68 | 0.63 | 0.74 |  |
| 36. When visiting a doctor or health provider I am able to tell him/her the name of the medications that I have previously used | 0.892 | 0.71 | 0.66 | 0.73 |  |
| 37. When visiting a doctor or health provider I am able to ask all the questions I have | 0.796 | 0.70 | 0.65 | 0.73 |  |
| 38. I can share the health information that I gather with others (e.g. family, friends, etc.) | 0.628 | 0.59 | 0.51 | 0.76 |  |
| 39. If I have any questions about health issues I am able to get information and advice from others | 0.611 | 0.58 | 0.50 | 0.76 |  |
| 40. When visiting a doctor or health provide I am able to ask questions based on my research | 0.679 | 0.65 | 0.58 | 0.74 |  |
| 41. I talk to my friends about avoiding risky behaviour (e.g. smoking, hookah, drugs, etc.) | 0.505 | 0.47 | 0.34 | 0.78 |  |
| ^a^Numeracy |  |  |  |  | 0.64 |
| F-HELMA Total Score |  |  |  |  | 0.89 |
| HLAT8 |  |  |  |  | 0.71 |

^a:^ Numeracy was not included in the factor loading because numeracy had a different scoring system.

Abbreviation: ICC =Intraclass coefficient; SD=standard deviation

**APPENDIX 2: F-HELMA questionnaire**

**Mesure des compétences en santé pour les adolescents (HELMA)**

Cher participant,

Ce questionnaire porte sur la perception que vous avez de vos capacités et compétences à obtenir et utiliser les informations relatives à la santé. Pour chaque question, veuillez cocher ou marquer d’une croix la case qui selon vous correspond au mieux à vos capacités ou vos compétences. Merci de répondre à toutes les questions.

| **Questions** | | **Jamais** | **Rarement** | | | **Parfois** | | **Souvent** | **Toujours** | |  |
| --- | --- | --- | --- | --- | --- | --- | --- | --- | --- | --- | --- |
| 1. J'essaie d'obtenir autant que possible davantage d’informations sur la santé | |  |  | | |  | |  |  | |  |
| 2. Je suis capable de trouver une information sur la santé dont j’ai besoin | |  |  | | |  | |  |  | |  |
| 3. Quand je suis malade ou que je rencontre des problèmes de santé, je peux obtenir les informations dont j'ai besoin | |  |  | | |  | |  |  | |  |
| 4. Je suis capable de demander aux autres les informations sur la santé dont j’ai besoin. | |  |  | | |  | |  |  | |  |
| 5. Je suis en mesure d'accéder aux informations concernant un régime alimentaire adéquat et sain pour ma tranche d'âge | |  |  | | |  | |  |  | |  |
| 6. Je suis capable d’accéder à des informations sur les activités physiques qui conviennent à ma tranche d’âge | |  |  | | |  | |  |  | |  |
| 7. Je suis capable d’accéder à des informations sur les soins de la peau et des cheveux qui sont appropriés et nécessaires pour ma tranche d'âge | |  |  | | |  | |  |  | |  |
| 8. Je suis capable d’accéder à des renseignements sur la santé mentale appropriés pour ma tranche d’âge | |  |  | | |  | |  |  | |  |
| 9. Je suis capable de trouver des sources d’information utiles à propos de la santé sur internet | |  |  | | |  | |  |  | |  |
| 10.Je peux lire des brochures à propos des médicaments prescrits | |  |  | | |  | |  |  | |  |
| 11. Je peux facilement lire des brochures éducatives sur des questions de nutrition | |  |  | | |  | |  |  | |  |
| 12. Je peux facilement lire des brochures / fiches d'information sur la prévention de maladies (par exemple l'anémie, l'ostéoporose, les infections respiratoires, etc.) | |  |  | | |  | |  |  | |  |
| 13. Je peux facilement lire des articles d’informations sur la santé dans les magazines et les journaux | |  |  | | |  | |  |  | |  |
| 14. Je peux facilement lire des documents d’information sur la santé sur Internet (ex : les sites web) | |  |  | | |  | |  |  | |  |
| **Questions** | | **Jamais** | **Rarement** | | | **Parfois** | | **Souvent** | **Toujours** | |  |
| 15. Je peux facilement comprendre la signification des signes utilisés dans les hôpitaux et centres médicaux. | |  |  | | |  | |  |  | |  |
| 16. Je peux comprendre la plupart des choses que j’entends au sujet de la santé | |  |  | | |  | |  |  | |  |
| 17. Je peux facilement comprendre le contenu des informations que je trouve sur la santé | |  |  | | |  | |  |  | |  |
| 18. Je peux facilement comprendre les instructions et les recommandations de mon médecin (ex : les ordonnances) | |  |  | | |  | |  |  | |  |
| 19. Je peux facilement comprendre les informations sur les médicaments : l’utilisation, les effets secondaires et les mises en garde | |  |  | | |  | |  |  | |  |
| 20. Je peux facilement comprendre les informations nutritionnelles sur les emballages des aliments | |  |  | | |  | |  |  | |  |
| 21. Je peux comprendre les informations et les recommandations sur une bonne nutrition pour les adolescents dans les médias (ex : la radio, la télévision, Internet, etc.) | |  |  | | |  | |  |  | |  |
| 22. Je peux comprendre les informations et les avertissements diffusés par les médias (par exemple à la radio, la télévision, Internet, etc.) sur le tabac, les drogues et les comportements à risque | |  |  | | |  | |  |  | |  |
| 23 Je peux comprendre les informations et les recommandations sur la santé et la maladie dans les médias |  | | |  |  | |  | | |  | |
| 24. Je peux comprendre les recommandations sur la prévention des accidents et des blessures. |  | | |  |  | |  | | |  | |
| 25. Face à de nouvelles informations sur la santé, je peux juger si elles sont justes ou pas |  | | |  |  | |  | | |  | |
| 26. Je compare les données obtenues de diverses sources |  | | |  |  | |  | | |  | |
| 27. Face à des informations contradictoires sur des questions de santé, je peux reconnaître l’information correcte |  | | |  |  | |  | | |  | |
| 28. J'ai la capacité de juger les sources auxquelles je peux faire confiance |  | | |  |  | |  | | |  | |
| 29. Lorsque je reçois des données sur la nutrition, je peux choisir la bonne information |  | | |  |  | |  | | |  | |
| **Questions** | **Jamais** | | | **Rarement** | **Parfois** | | **Souvent** | | | **Toujours** | |
| 30. Lorsque je fais mes courses, je choisis un aliment en fonction des données nutritionnelles inscrites sur son emballage (ex : quantité de sucre, d’énergie, de protéines, etc.) |  | | |  |  | |  | | |  | |
| 31. J'essaie de choisir des aliments sans conservateurs |  | | |  |  | |  | | |  | |
| 32. J’essaie d’appliquer ce que j’ai appris sur les questions de santé dans ma vie de tous les jours |  | | |  |  | |  | | |  | |
| 33. J’essaie de maintenir un poids équilibré |  | | |  |  | |  | | |  | |
| 34.Je peux discuter de mes préoccupations liées aux questions de santé avec des agents de santé |  | | |  |  | |  | | |  | |
| 35. Lorsque je consulte un médecin ou un agent de santé, je suis capable de lui donner tous mes renseignements personnels nécessaires |  | | |  |  | |  | | |  | |
| 36. Lorsque je consulte un médecin ou un agent de santé, je suis capable de lui donner le nom des médicaments que j’ai pris par le passé |  | | |  |  | |  | | |  | |
| 37. Lorsque je consulte un médecin ou un agent de santé, je suis capable de lui poser toutes les questions que j’ai. |  | | |  |  | |  | | |  | |
| 38. Je peux partager les informations sur la santé que je recueille avec d’autres personnes (ex : ma famille, mes amis, etc.) |  | | |  |  | |  | | |  | |
| 39. Si j’ai n’importe quelle question sur la santé, je suis capable d’obtenir des informations et conseils venant des autres. |  | | |  |  | |  | | |  | |
| 40. Lorsque je consulte un médecin ou un agent de santé, je suis capable de lui poser des questions basées sur les recherches que j’ai effectuées |  | | |  |  | |  | | |  | |
| 41. Je parle à mes amis afin qu’ils évitent les comportements à risque (par ex : la cigarette, la chicha, la drogue etc.) |  | | |  |  | |  | | |  | |

42. Les informations suivantes sont inscrites sur l’emballage d'une boîte de lait. Si une personne boit 3 tasses de lait en une journée, combien de glucides a-t-elle consommée ?

| **Valeurs nutritionnelles** |
| --- |
| **Portion** : 1 tasse (240 cc) |
| **Portions par boîte**: 4 |
| **Quantités par portion** : |
| **Energie** : 140 kcal |
| **Lipides** : 7g**Cholestérol** : 30 mg |
| **Glucides** : 11 g  **Sucre** : 0 g |
| **Protéines** : 8 g |
| **Sodium** : 160 mg |

43. Calculez l’IMC (Indice de Masse Corporelle) d’une personne ayant une taille = 160 cm et un poids = 70 kg

**IMC=** $\frac{poids(kg)}{taille{(m)}^{\boldsymbol{2}}}$

44. Selon les informations suivantes, quelle est la corpulence de cette personne ?

|  | **Insuffisance pondérale** | **Poids normal** | **Surpoids** | **Obèse** |
| --- | --- | --- | --- | --- |
| **IMC** | **<18.5** | **18.5** ‒ **24.9** | **25** ‒ **29.9** | **≥30** |

**a**- Insuffisance pondérale (poids insuffisant) **b**- Poids normal

**c**-Surpoids **d**- Obèse

**APPENDIX 3**

Descriptive Statistics of the 7-Factor model of the F-HELMA

| **Items** | **Mean** | **SD** | **IQR** |
| --- | --- | --- | --- |
| **Self-Efficacy** |  |  |  |
| 1. I try to get more information about health as much as possible | 3.02 | 1.29 | 2.00 |
| 2. I am able to find health information that I need | 3.37 | 1.23 | 2.00 |
| 3. When ill or facing health problems. I can get the necessary information I need | 3.52 | 1.28 | 3.00 |
| 4. I am able to ask others about health information that I need | 3.46 | 1.30 | 3.00 |
| **Access** |  |  |  |
| 5. I am able to access information about the healthy diet that is appropriate for my age group | 3.05 | 1.29 | 2.00 |
| 6. I am able to access information about the physical activity appropriate for my age group | 3.23 | 1.25 | 2.00 |
| 7. I am able to access information about the proper care required for my skin and hair that is appropriate for my age group | 3.10 | 1.35 | 2.00 |
| 8. I am able to access information about mental health appropriate for my age group | 2.98 | 1.31 | 2.00 |
| 9. I am able to find useful resources about health Information on the Internet | 3.33 | 1.30 | 2.00 |
| **Reading** |  |  |  |
| 10. I can read brochures on prescribed medicine | 3.29 | 1.24 | 2.00 |
| 11. I can easily read educational brochures about nutritional issues | 3.60 | 1.32 | 2.00 |
| 12. I can easily read brochures/fact sheets about disease prevention (e.g. anemia. osteoporosis. respiratory infections. etc.) | 3.16 | 1.26 | 2.00 |
| 13. I can easily read health information materials in magazines and newspapers | 2.96 | 1.28 | 2.00 |
| 14. I can easily read health information materials on the Internet (e.g. websites) | 3.24 | 1.26 | 2.00 |
| **Understanding** |  |  |  |
| 15. I can easily understand the meaning of the signs used in hospitals and medical centers | 2.45 | 1.22 | 2.00 |
| 16. I can understand most things I hear about health | 3.18 | 1.14 | 2.00 |
| 17. I can easily understand the content of health information that I find | 3.04 | 1.15 | 2.00 |
| 18. I can easily understand my doctor’s instructions and recommendations (e.g. prescriptions) | 3.68 | 1.29 | 2.00 |
| 19. I can easily understand information about medications – usage. side effects and warnings | 3.51 | 1.29 | 3.00 |
| 20. I can easily understand the nutrition facts on food packages | 3.37 | 1.18 | 2.00 |
| 21. I can understand the information and recommendations about proper nutrition for adolescents in the media (e.g. radio. TV. internet. etc.) | 3.32 | 1.24 | 2.00 |
| 22. I can understand the information and warnings provided by the media (e.g. radio. TV. internet. etc.) about tobacco, drug abuse and risky behaviors | 3.67 | 1.25 | 2.00 |
| 23. I can understand the information and recommendations about health and illness in the media | 3.46 | 1.10 | 1.00 |
| 24. I can understand the recommendations on prevention of accidents and injuries | 3.40 | 1.21 | 1.00 |
| **Appraisal** |  |  |  |
| 25. When faced with new health information, I can judge its accuracy | 3.03 | 1.24 | 2.00 |
| 26. I would compare the data obtained from various sources | 2.82 | 1.31 | 2.00 |
| 27. When dealing with conflicting information about health issues. I can recognize the correct information | 2.96 | 1.15 | 2.00 |
| 28. I have the ability to judge which resources I can trust | 3.20 | 1.21 | 2.00 |
| 29. When dealing with nutritional information I can choose the right information | 3.55 | 1.24 | 2.00 |
| **Use -** |  |  |  |
| 30. When shopping, I choose food based on its nutrition facts (e.g. amount of energy, sugar, protein, etc.) written on the packaging | 2.49 | 1.20 | 1.00 |
| 31. I try to choose foods without preservatives | 2.56 | 1.43 | 3.00 |
| 32. I try to apply what I have learned about health issues in my everyday life | 3.30 | 1.23 | 2.00 |
| 33. I try to keep my body weight in balance | 3.29 | 1.32 | 2.00 |
| **Communicate -** |  |  |  |
| 34. I can discuss my concerns relating to health issues with health providers | 3.03 | 1.34 | 2.00 |
| 35. When visiting a doctor or health provider I am able to give him/her all of my necessary personal information | 3.25 | 1.33 | 2.00 |
| 36. When visiting a doctor or health provider I am able to tell him/her the name of the medications that I have previously used | 3.57 | 1.32 | 2.00 |
| 37. When visiting a doctor or health provider I am able to ask all the questions I have | 3.62 | 1.34 | 3.00 |
| 38. I can share the health information that I gather with others (e.g. family, friends, etc.) | 3.64 | 1.20 | 2.00 |
| 39. If I have any questions about health issues, I am able to get information and advice from others | 3.43 | 1.18 | 1.00 |
| 40. When visiting a doctor or health provide, I am able to ask questions based on my research | 3.19 | 1.29 | 2.00 |
| 41. I talk to my friends about avoiding risky behavior (e.g., smoking, hookah, drugs, etc.) | 3.82 | 1.25 | 2.00 |
| F-HELMA Total Score | 54.82 | 14.28 | 19.70 |
| HLAT8 | 22.75 | 6.75 | 8.00 |

F-HELMA scores range from 0 to 100 with higher scores indicating higher health literacy level: HLAT-8 scores range from 1 to 37 with higher scores indicating higher health literacy levels: SD, standard deviation: IQR, Interquartile Range
